# Avionic Air Data Sensors Fault Detection and Isolation by means of Singular Perturbation and Geometric Approach

**DOI:** 10.3390/s17102202

**Published:** 2017-09-25

**Authors:** Paolo Castaldi, Nicola Mimmo, Silvio Simani

**Affiliations:** 1 Department of Electrical, Electronic and Information Engineering, University of Bologna, Faculty of Aerospace Engineering, Via Fontanelle 40, 47121 Forlí, Italy; paolo.castaldi@unibo.it; 2 Department of Engineering, University of Ferrara, Via Saragat 1, 44122 Ferrara, Italy; silvio.simani@unife.it

**Keywords:** singular perturbation, NonLinear Geometric Approach, fault detection and isolation, aircraft, autopilot avionics, air data sensors

## Abstract

Singular Perturbations represent an advantageous theory to deal with systems characterized by a two-time scale separation, such as the longitudinal dynamics of aircraft which are called *phugoid* and *short period*. In this work, the combination of the NonLinear Geometric Approach and the Singular Perturbations leads to an innovative Fault Detection and Isolation system dedicated to the isolation of faults affecting the air data system of a general aviation aircraft. The isolation capabilities, obtained by means of the approach proposed in this work, allow for the solution of a fault isolation problem otherwise not solvable by means of standard geometric techniques. Extensive Monte-Carlo simulations, exploiting a high fidelity aircraft simulator, show the effectiveness of the proposed Fault Detection and Isolation system.

## 1. Introduction

Current manned and unmanned aircraft implement autopilots ranging from simple stability augmentation systems to complex navigation systems. Due to the high level of criticality of aircraft, autopilots need to implement robust design strategies to guarantee, for example, appropriate levels of fault tolerance. In particular, a review of aeronautical worldwide sources on safety event reports reveals repeated instances of anomalous Air Data Sensor (ADS) events. The nature of ADS measurements, indeed, makes air-data probes subject to the spectrum of environmental conditions and, even with a design meant to withstand harsh conditions, instances of ADS probe faults have been recorded for diverse platform types and situations [1,2,3] and citations therein.

The Fault Detection and Isolation (FDI) represents a relevant and critical theme in many sectors such as surgery [4], automotive [5], electrical engineering [6], wind turbines [7] and aerospace engineering [8,9,10,11,12,13,14,15,16,17,18]. Although aircraft subsystems are designed with sufficient levels of redundancy to tolerate both hardware and software faults, FDI algorithms are suitable to monitor the aircraft safety and assess the eventual presence of anomalies, [19,20,21,22,23]. The main goal of the FDI is represented by the decision about the status of health of the system (fault detection) and, in case of faults, the second objective is the localization as well as the determination of the fault nature [24]. Furthermore, the importance and relevance of Air Data Sensor FDI has been highlighted by the European project (ADDSAFE, Advanced Fault Diagnosis for Sustainable Flight Guidance and Control) which was focused on the study of this problem [25].

Common FDI systems are based on either hardware or analytical redundancies but, in recent years, the aerospace industry is demanding for solutions guaranteeing the same or an improved fail safety level with a reduced level of hardware redundancy [26]. From one hand both general aviation aircraft and UAVs have not the payload capacity to be equipped with standard hardware redundancy and, on the other hand, engineers designing large aircraft try to save weight to reduce the consumed fuel, costs, noise and pollution. Conversely, analytical redundancy does not require additional hardware because exploits the mathematical model of the system as redundant information but is more challenging due to the need of guaranteeing its robustness in the presence of unknown disturbances, noise and model uncertainties.

**Figure 1:**
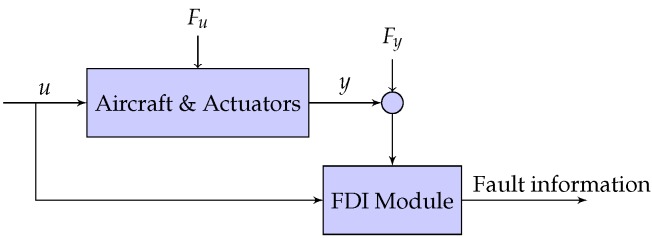
Representation of the system affected by actuator and sensor faults.

Different model based FDI methods have been developed during recent years [27,28,29,30] but all of them share the same common structure highlighted in Figure 1. The state of art sees model based FDI methodologies be roughly classified in two main groups: those based on a bank of detection residuals each one trivially dependent on different fault components and those which implement a fault re-constructor. With reference to the FDI methods belonging to the first group, this work introduces an innovative approach applicable to those systems which can be described by a singular perturbation model.

This paper presents a model based FDI approach which solves the problem with a two-step procedure. In the first phase a set of variables known as residuals is provided by one or more parallel residual generators: each residual is designed to be zero (or zero mean) in absence of faults belonging to a certain different subset of the whole faults set. The second step is represented by the decision, made on the base of the analysis of the residuals, about the presence of faults (fault detection) and which is the fault that is affecting the system (fault isolation). The FDI module proposed in this paper is designed by means of the NonLinear version of the Geometric Approach (NLGA) [31,32]. Thanks to the NLGA it is possible to design detection residuals which are insensitive to a selected subset of all the faults potentially affecting the system. The fault isolation is obtained by collecting the detection residuals, which are insensitive to different fault subsets, in a bank of residuals whose configuration identifies which fault is present.

This paper proposes, for the design of the residual generators, an innovative and combined use of the NonLinear version of the Geometric Approach (NLGA) [31,32] and of the Singular Perturbations (SP) theory [33]. This work shows that this kind of strategy allows for the solution of the fault isolation problem, for general aviation aircraft affected by faults on air data sensors, not otherwise solvable with the standard NLGA approach, see [31,34].

Thanks to the NLGA it is possible to analyse the system to identify the isolable faults. The results of this analysis are valid for any possible methodological tool successively exploited for the design of the detection residuals. When two or more faults are unisolable the only methodological approach which can provide information about the presence of these faults consists in an estimation of the whole unisolable fault vector thus requiring the implementation of high order input re-constructor systems. With the goals of reducing the complexity of the FDI system, to increase its modularity and to make it distributable, the designers try to minimize the estimator dimensions. The literature presents some remarkable works dealing with the detection and the isolation of faults affecting aircraft air data such as [1,2,3,10,13,17,20,21,35] in which the detection filters are base on full order unknown input re-constructors. At the opposite, this paper focuses on the reduction of dimensions of the unisolable fault set and, thanks to the innovative combined use of the SP and the NLGA, allows for the isolation of the angle of attack faults, otherwise not possible, by means of reduced order detection filters.

The paper is organized as follows: Section 2 describes the nonlinear aircraft model used for the FDI module design. Section 3 details the Air Data System and its associated fault scenario whereas Section 4 proposes the design of the novel FDI module, based on the NLGA combined with SP theory. Finally, in Section 5 simulations results, based on a general aviation aircraft flight simulator and Monte-Carlo simulations, are given, showing the effectiveness of the approach and the good performance of the FDI system.

## 2. Nonlinear Longitudinal Aircraft Model

In this paper the aircraft is modelled as a rigid body subject to the following external forces and momentums: gravity, aerodynamics and engine thrust. The longitudinal dynamics of aircraft can be represented by the following equations, [36]:

(1) $$\begin{array}{ccc} \dot{h} & = & v_{c}\\ \dot{V} & = & \frac{T}{m}\cos\alpha -\frac{q_{d}SC_{D_{TOT}}}{m}-g\frac{v_{c}}{V}\\ {\dot{v}}_{c} & = & \frac{T}{m}\left(\cos\alpha \frac{v_{c}}{V}+\sin\alpha \sqrt{1-{\left(\frac{v_{c}}{V}\right)}^{2}}\right)-g+\frac{q_{d}SC_{L_{TOT}}}{m}\sqrt{1-{\left(\frac{v_{c}}{V}\right)}^{2}}-\frac{q_{d}SC_{D_{TOT}}}{m}\frac{v_{c}}{V}\\ \dot{\alpha } & = & q-\frac{1}{mV}\left(T\sin\alpha +q_{d}SC_{L_{TOT}}-mg\sqrt{1-{\left(\frac{v_{c}}{V}\right)}^{2}}\right)\\ \dot{q} & = & \frac{1}{I_{y}}\left(d_{T}T+q_{d}S\bar{c}C_{m_{TOT}}\right) \end{array}$$

where *h* is the altitude, *V* is the airspeed, $v_{c}$ the rate of climb, α the angle of attack and *q* is the pitch rate. The term *m* indicates the mass, $I_{y}$ the longitudinal inertia, $q_{d}$ is the dynamic pressure, *S* is the wing reference area and $\bar{c}$ indicates the mean aerodynamics chord, *g* represents the gravity acceleration and $d_{T}$ the thrust arm. The aerodynamic forces and momentums coefficients are $C_{L_{TOT}}$, $C_{D_{TOT}}$ and $C_{m_{TOT}}$. Finally, *T*, indicating the thrust, is the first control input. For a conventional aircraft, the typical component build–up technique, see [36], leads to the following standard aerodynamics description:

(2) $$\begin{array}{ccc} C_{D_{TOT}} & = & C_{D_{0}}+C_{D_{\alpha }}\alpha \\ C_{L_{TOT}} & = & C_{L_{0}}+C_{L_{\alpha }}\alpha +C_{L_{q}}\frac{q\bar{c}}{2V}+C_{L_{\delta_{e}}}\delta_{e}\\ C_{m_{TOT}} & = & C_{m_{0}}+C_{m_{\alpha }}\alpha +C_{m_{q}}\frac{q\bar{c}}{2V}+C_{m_{\delta_{e}}}\delta_{e} \end{array}$$

where the aerodynamics coefficients $C_{s_{k}}$ with s∈{D,L,m} and $k\in \{0,\alpha ,q,\delta_{e}\}$ are considered known parameters thanks to wind tunnel and flight tests. The term $\delta_{e}$, representing the elevator deflection, is the second control input. The overall model can be rewritten in the following compact form:

(3) $$\left[\begin{array}{c} {\dot{x}}_{1}\\ \varepsilon {\dot{x}}_{2} \end{array}\right]=\left[\begin{array}{c} g_{1,0}\left(x_{1},x_{2}\right)\\ g_{2,0}\left(x_{1},x_{2}\right) \end{array}\right]+\sum_{i=1}^{p}\left[\begin{array}{c} g_{1,i}\left(x_{1},x_{2}\right)\\ g_{2,i}\left(x_{1},x_{2}\right) \end{array}\right]u_{i}$$

where $x_{1}={\left[h,V,v_{c}\right]}^{T}\in R^{n_{1}}$ and $x_{2}={\left[\alpha ,q\right]}^{T}\in R^{n_{2}}$ represent the state vector, suitably partitioned, and where the term, ε, is the small positive perturbation parameter, called *singular*. Moreover, the actuator inputs are two, i.e., p=2, with $u_{1}=T$ and $u_{2}=\delta_{e}$. The model is completed by the outputs $y_{1}=x_{1}$ and $y_{2}=x_{2}$ whereas the non linear term $g_{i,j}\left(x_{1},x_{2}\right)$ with i∈{1,2} and j∈{0,⋯,p} are:

(4) $$g_{1,0}\left(x_{1},x_{2}\right)=\left[\begin{array}{c} v_{c}\\ -\frac{q_{d}SC_{D_{TOT}}}{m}-g\frac{v_{c}}{V}\\ \frac{q_{d}S\left(C_{L_{0}}+C_{L_{\alpha }}\alpha +C_{L_{q}}\frac{q\bar{c}}{2V}\right)}{m}\sqrt{1-{\left(\frac{v_{c}}{V}\right)}^{2}}-\frac{q_{d}SC_{D_{TOT}}}{m}\frac{v_{c}}{V}-g \end{array}\right]$$

(5) $$\begin{array}{c} g_{2,0}\left(x_{1},x_{2}\right)=\left[\begin{array}{c} q-\frac{1}{mV}\left(q_{d}S\left(C_{L_{0}}+C_{L_{\alpha }}\alpha +C_{L_{q}}\frac{q\bar{c}}{2V}\right)-mg\sqrt{1-{\left(\frac{v_{c}}{V}\right)}^{2}}\right)\\ \frac{1}{I_{y}}q_{d}S\bar{c}\left(C_{m_{0}}+C_{m_{\alpha }}\alpha +C_{m_{q}}\frac{q\bar{c}}{2V}\right) \end{array}\right] \end{array}$$

(6) $$g_{1,1}\left(x_{1},x_{2}\right)=\left[\begin{array}{c} 0\\ \frac{\cos\alpha }{m}\\ \frac{1}{m}\left(\cos\alpha \frac{v_{c}}{V}+\sin\alpha \sqrt{1-{\left(\frac{v_{c}}{V}\right)}^{2}}\right) \end{array}\right]\;g_{2,1}\left(x_{1},x_{2}\right)=\left[\begin{array}{c} -\frac{\sin\alpha }{mV}\\ \frac{d_{T}}{I_{y}} \end{array}\right]$$

(7) $$g_{1,2}\left(x_{1},x_{2}\right)=\left[\begin{array}{c} 0\\ 0\\ \frac{1}{m}q_{d}S\sqrt{1-{\left(\frac{v_{c}}{V}\right)}^{2}}C_{L_{\delta_{e}}} \end{array}\right]\;g_{2,2}\left(x_{1},x_{2}\right)=\left[\begin{array}{c} -\frac{1}{mV}q_{d}SC_{L_{\delta_{e}}}\\ \frac{1}{I_{y}}q_{d}S\bar{c}C_{m_{\delta_{e}}} \end{array}\right]$$

**Hypothesis** **1.** *AutoPilot Control model: in general, the design of the FDI module should be independent from the structure of the controller except for some high level properties introduced by means of the control unit, such as the stability of the closed loop system. In detail, this work proposes an FDI scheme which can be successfully applied to flight controllers which have sufficiently slow outputs to allow for the definition of the manifold $x_{2_{M}}$ which will be defined later. In particular:*

- given the state $x={\left[x_{1}^{T},\;x_{2}^{T}\right]}^{T}\in X\subset R^{n}$, the control laws $u_{i}$ are sufficiently smooth functions of the states x, i.e., $u_{i}=u_{i}\left(x\right)$, and there exist a Lipschitz constant L such that $\left|\right|u_{i}\left(x^{⋆}\right)-u_{i}\left(x\right)\left||<L|\right|x^{⋆}-x\left|\right|$$\forall \;x,x^{⋆}\in X$;
- the control laws $u_{i}\left(x\right)$ are built up by two components $u_{i}\left(x\right)=u_{i}^{f}\left(x\right)+u_{i}^{s}\left(x\right)$ where $u_{i}^{f}\left(x\right)$ represents the fast component and $u_{i}^{s}\left(x\right)$ is the slow contribution;
- *the fast term $u_{i}^{f}\left(x\right)$ is not active when $\varepsilon {\dot{x}}_{2}=0$, i.e., it is such that*
  $$0=g_{2,0}\left({\bar{x}}_{1},x_{2}\right)+\sum_{i=1}^{p}g_{2,i}\left({\bar{x}}_{1},x_{2}\right)u_{i}^{s}\left(x\right);$$
- *the slow control law, $u_{i}^{s}\left(x\right)$, is such that*
  $$\lim_{\varepsilon \to 0}\varepsilon \frac{d}{dt}u_{i}^{s}\left(x\right)=0.$$

The assumption of having ε=0 shrinks the state-space dimension from $n_{1}+n_{2}$ to $n_{1}$ because of the degeneration to an algebraic equation of the second relation in (3). In this slow time scale the fast state, $x_{2}$, evolve on a manifold defined by the approximated slow state $x_{1}$ and the slow part of the control law, $u_{i}^{s}$, respectively identified by ${\bar{x}}_{1}$ and ${\bar{u}}_{i}^{s}$:

(8) $$\left[\begin{array}{c} {\dot{\bar{x}}}_{1}\\ 0 \end{array}\right]=\left[\begin{array}{c} g_{1,0}\left({\bar{x}}_{1},x_{2}\right)\\ g_{2,0}\left({\bar{x}}_{1},x_{2}\right) \end{array}\right]+\sum_{i=1}^{p}\left[\begin{array}{c} g_{1,i}\left({\bar{x}}_{1},x_{2}\right)\\ g_{2,i}\left({\bar{x}}_{1},x_{2}\right) \end{array}\right]{\bar{u}}_{i}^{s}$$

Equations (8) represent the *reduced model*.

For standard aircraft, the algebraic system in (8)

(9) $$0=g_{2,0}\left({\bar{x}}_{1},x_{2}\right)+\sum_{i=1}^{p}g_{2,i}\left({\bar{x}}_{1},x_{2}\right){\bar{u}}_{i}^{s}$$

admits one isolated root, $x_{2_{M}}=M\left({\bar{x}}_{1},{\bar{u}}_{1}^{s},\cdots ,{\bar{u}}_{p}^{s}\right)$.

On the other hand, during the transient phases of the fast variables, the SP theory treats the slow variables as parameters. Defined the time scale change, $\tau =\left(t-t_{0}\right)/\varepsilon$ with $t_{0}>0$, the apex derivatives are defined as $x^{\prime }=dx/d\tau$ and the dynamic order of the system is reduced from $n_{1}+n_{2}$ to $n_{2}$ by assuming ε=0:

(10) $$\left[\begin{array}{c} x_{1}^{\prime }\\ x_{2}^{\prime } \end{array}\right]=\left[\begin{array}{c} 0\\ g_{2,0}\left(x_{1},x_{2}\right) \end{array}\right]+\sum_{i=1}^{p}\left[\begin{array}{c} 0\\ g_{2,i}\left(x_{1},x_{2}\right) \end{array}\right]u_{i}$$

Equations (10) represent the *boundary–layer model*. Defined the error $z_{2}=x_{2}-x_{2_{M}}$, the approximated model is well posed if the following hypothesis is verified [33]:

**Hypothesis** **2.** *The fast control law, $u_{i}^{f}$, is such that the origin of the system*

(11) $$\begin{array}{ccc} \frac{d}{d\tau }z_{2} & = & g_{2,0}\left({\bar{x}}_{1},x_{2_{M}}+z_{2}\right)+\sum_{i=1}^{p}g_{2,i}\left({\bar{x}}_{1},x_{2_{M}}+z_{2}\right)\left({\bar{u}}_{i}^{s}+u_{i}^{f}\right) \end{array}$$

*is a locally exponential stable equilibrium point, uniformly in $\left({\bar{x}}_{1},{\bar{u}}_{i}^{s}\right)$.*

**Lemma** **1.** *Given two matrices $K_{1}$ and $K_{2}$ with proper dimensions, any linear static state feedback control law $u_{i}\left(x\right)=K_{1}x_{1}+K_{2}\left(x_{2}-x_{2_{M}}\right)$ verifies the Hypotheses 1 and 2.*

**Proof.**  Let us define the fast control law with $u^{f}=K_{2}\left(x_{2}-x_{2_{M}}\right)$ and the slow control law with $u_{i}^{s}=K_{1}x_{1}$. The slow control law is such that $\lim_{\varepsilon \to 0}\varepsilon \frac{d}{dt}u_{i}^{s}=K_{1}\lim_{\varepsilon \to 0}\varepsilon \frac{d}{dt}x_{1}=K_{1}x_{1}^{\prime }=0$. On the other hand, the fast control law is null when $x_{2}=x_{2_{M}}$ i.e., when $0=g_{2,0}\left({\bar{x}}_{1},x_{2}\right)+\sum_{i=1}^{p}g_{2,i}\left({\bar{x}}_{1},x_{2}\right){\bar{u}}_{i}^{s}$. Finally, the linearisation of the system (11) at the the origin is represented by ${\dot{z}}_{2}=Az_{2}+\sum_{i=1}^{p}b_{i}u_{i}$ where the couple $\left(A,\left[b_{1},\cdots ,b_{p}\right]\right)$, for standard aircraft configurations, is stabilizable thus implying the existence of a state feedback matrix $K_{2}$ such that the origin of the system is locally exponentially stable. ☐

**Remark** **1.** Thanks to the state augmentation technique, the results of Lemma 1 can be extended to linear dynamic controllers to demonstrate that standards autopilots installed on general aviation aircraft, usually implementing proportional–integral–derivative structures, fulfil the Hypotheses 1 and 2.

Finally, the actual system dynamics (3) can be approximated by:

(12) $$\begin{array}{ccc} \left[\begin{array}{c} {\dot{\bar{x}}}_{1}\\ x_{2}^{\prime } \end{array}\right] & = & \left[\begin{array}{c} g_{1,0}\left({\bar{x}}_{1},M\left({\bar{x}}_{1},{\bar{u}}_{1}^{s},\cdots ,{\bar{u}}_{p}^{s}\right)\right)\\ g_{2,0}\left({\bar{x}}_{1},x_{2}\right) \end{array}\right]+\sum_{i=1}^{p}\left[\begin{array}{c} g_{1,i}\left({\bar{x}}_{1},M\left({\bar{x}}_{1},{\bar{u}}_{1}^{s},\cdots ,{\bar{u}}_{p}^{s}\right)\right)\\ g_{2,i}\left({\bar{x}}_{1},x_{2}\right) \end{array}\right]u_{i} \end{array}$$

## 3. Air Data System

**Figure 2:**
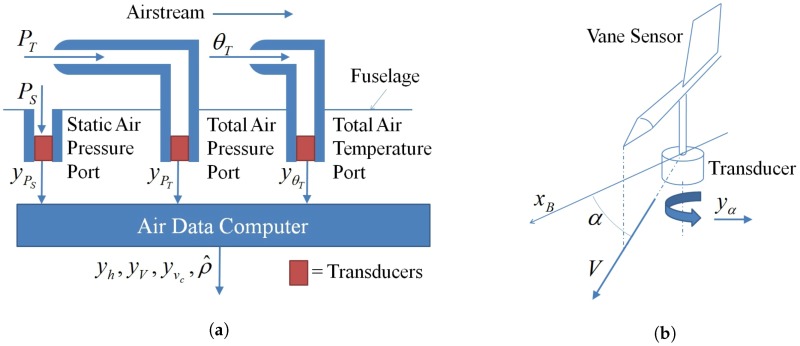
Air Data System: (**a**) Computing of the air data; (**b**) Angle of Attack Measurement Subsystem: the axis $x_{B}$ is a body fixed reference frame.

The Air Data System (ADS) is one of the fundamental subsystems of aircraft because, thanks to it, autopilots can regulate the airspeed, *V*, and barometric altitude, *h*, which are necessary for guaranteeing the stability and for the guidance of the air-plane. Standard ADSs also include the measurement of the angle of attack, α, extremely useful for avoiding instantaneous dangerous stall situations (for example due to aggressive manoeuvres or unexpected wind). ADSs are composed by two main parts: the probes and the computational unit, see Figure 2a. The probes are constituted by a “total air pressure” port, a “static air pressure” port, a “total air temperature” port and a “vane sensor” which provide the measurements $y_{P_{T}}$, $y_{P_{S}}$, $y_{\theta_{T}}$ and $y_{\alpha }$ respectively.

The “total air pressure”, $P_{T}$, and the “static air pressure”, $P_{S}$, are linked to the airspeed *V* thanks to the Bernoulli’s law:

(13) $$P_{T}=P_{S}+\frac{1}{2}\rho V^{2}$$

where ρ indicates the air density, in turn linked to the static air temperature, $\theta_{S}$, and pressure, $P_{S}$, by means of the ideal gas law:

(14) $$P_{S}=\rho R\theta_{S}$$

where *R* is the ideal gas constant for air R=287.04 m^2^/(°C s^2^). Finally the International Standard Atmosphere (ISA) model [37] introduced a mathematical description of the static air pressure, $P_{S}$ as function of the geometric height above the mean sea level, *H*:

(15) $$\begin{array}{ccc} P_{S_{ISA}}\left(H\right) & = & P_{0}{\left(1-KH\right)}^{\frac{g}{RK}} \end{array}$$

where $P_{0}=101,325$ Pa represents the standard static pressure at the seal level and $K=4.193\times 10^{-4}$ m^−1^ is the temperature rate in the troposphere.

To correctly compute the barometric altitude, the airspeed *V* and the rate of climb $v_{c}$, the total air temperature $\theta_{T}$ is exploited to determine, combined with the static air pressure the air density. These computations are usually implemented into an Air Data Computer (ADC) which receive $y_{P_{T}}$, $y_{P_{S}}$, $y_{\theta_{T}}$ and $y_{\alpha }$ and give as output the estimated barometric altitude $y_{h}$, the estimated airspeed $y_{V}$ and the estimated rate of climb $y_{v_{c}}$:

(16) $$\begin{array}{ccc} y_{h} & = & P_{S_{ISA}}^{-1}\left(y_{P_{S}}\right)\\ \hat{M} & = & \sqrt{\frac{2}{\gamma -1}\left[{\left(\frac{y_{P_{T}}}{y_{P_{S}}}\right)}^{\frac{\gamma -1}{\gamma }}-1\right]}\\ {\hat{\theta }}_{S} & = & \frac{y_{\theta_{T}}}{1+\frac{\gamma -1}{2}{\hat{M}}^{2}} \end{array}\;\begin{array}{ccc} y_{V} & = & \sqrt{\gamma R{\hat{\theta }}_{S}}\hat{M}\\ y_{v_{c}} & = & \frac{\partial P_{S_{ISA}}^{-1}\left(y_{p_{s}}\right)}{\partial P_{S}}dy_{P_{S}}\\ {\dot{dy}}_{P_{S}} & = & \lambda_{P_{S}}\left({\dot{y}}_{P_{S}}-dy_{P_{S}}\right) \end{array}$$

where $\hat{M}$ indicates the Mach number estimation, ${\hat{\theta }}_{S}$ the static air temperature estimation and γ is the ratio of specific heats. The term $dy_{P_{S}}$ represents a low-pass filtered version of ${\dot{y}}_{P_{S}}$ where $\lambda_{P_{S}}$ is the pole of the filter. Finally, the FDI module needs of an estimation of the air density, $\hat{\rho }$, which is provided by the ADS by means of the following equation:

(17) $$\hat{\rho }=\frac{y_{P_{S}}}{R{\hat{\theta }}_{S}}$$

The angle of attack, α, represents a crucial quantity which describes the aerodynamic behaviour of the aircraft in each flight condition. The aerodynamic lift, *L*, is directly (quasi-linearly) proportional to the angle of attack until α remains in certain limits, $\alpha \in [\alpha_{min}^{lin},\alpha_{max}^{lin}]$, determined by the aircraft shape. Close these limits the lift the aircraft can produce increases but non linearly and reach a (minimum)maximum at $\left(\alpha_{min}^{L}\right)\alpha_{max}^{L}$. Further increases of α correspond to a decrease of *L* up to the so called “stall angle” $\alpha^{stall}$ at which the lift suddenly decreases to zero. The autopilot has to guarantee that α always remains in certain predetermined safety regions of the open set $\left(\alpha_{min}^{stall},\alpha_{max}^{stall}\right)$. Usually the angle of attack is measured by means of vane sensors constituted by a mechanical flag which align itself with the air-stream and a electronic transducer which provides the measurement of the rotation angle $y_{\alpha }$, see Figure 2b.

### Air Data System Faults

**Figure 3:**
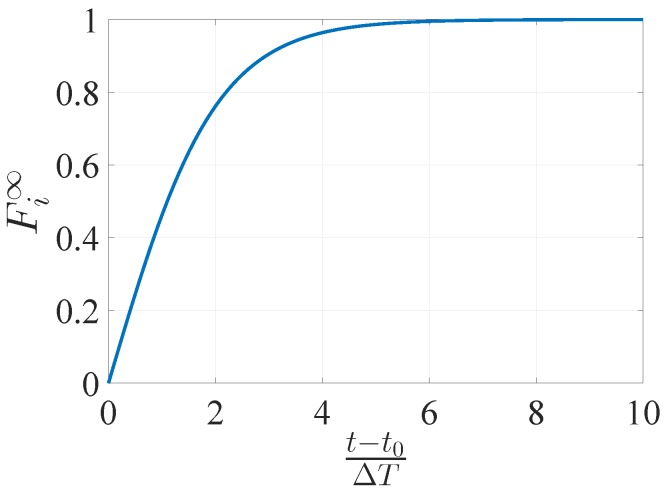
Fault transient behavior.

This work demonstrates that, thanks to the joint use of the NLGA and the SP, faults affecting ADS can be correctly detected and isolated. To this end, the model of the system, even in presence of such faults, have to hold the singular perturbation properties. It is worth observing that failures on sensors which induce the instability of aircraft are not taken into account because no hardware redundancy is considered in this work. Different strategies, which are out of the scope of this paper, can be exploited to deal with this kind of fault scenario. Furthermore, only multiple (more than one) but non concurrent (only one per time) faults are considered in this work. The *i*-th fault is indicated by $F_{i}$ where $F_{i}\left(x,u,t,t_{0}\right)\to R$ is a scalar real values function dependent on time, input and state. The faults affect the system at time $t_{0}\in R$, with $t_{0}>0$, so that $F_{i}\left(x,u,t,t_{0}\right)=0$ $\forall t<t_{0}$ and progressively reach their asymptotic value $F_{i}^{\infty }$ (see Figure 3)

(18) $$F_{i}\left(x,u,t,t_{0}\right)=F_{i}^{\infty }\frac{1-e^{-\left(t-t_{0}\right)/\Delta_{t}}}{1+e^{-\left(t-t_{0}\right)/\Delta_{t}}}\;\forall t\geq t_{0}$$

where $\Delta_{t}\in R_{>0}$ is a time constant. The subscript $i\in \{1,\cdots ,n_{F}\}$ indicates the physical faults on altitude, air speed, rate of climb and angle of attack sensor. Finally, to improve the readability of this manuscript, the dependencies of faults are omitted but where necessary.

The faults are modelled in terms of variation, from the nominal behaviour, due to biases and sensitivity modifications as

(19) $$y_{s}=s+F_{s}\;s=\left\{P_{S},P_{T},\theta_{T},\alpha \right\}$$

where $F_{s}=b_{s}+\left(k_{s}-1\right)s$, $\left(b_{s},k_{s}\right)\in R\times R_{+}$. In particular, the faulty static and total pressure outputs are described by:

(20) $$\begin{array}{ccc} y_{P_{T}} & = & P_{S}+\frac{1}{2}\rho V^{2}+F_{P_{T}}\\ y_{P_{S}} & = & P_{S}+F_{P_{S}}\\ y_{\theta_{T}} & = & \theta_{S}\left(1+\frac{\gamma -1}{2}\gamma R\theta_{S}V^{2}\right)+F_{\theta_{T}} \end{array}$$

where $F_{P_{T}}$, $F_{P_{S}}$ and $F_{\theta_{T}}$ respectively indicate the faults affecting the total pressure, the static pressure and the total temperature port. The faults influencing both pressure and temperature ports result in faults on altitude, $F_{h}$, airspeed, $F_{V}$, and rate of climb, $F_{v_{c}}$, as:

(21) $$\begin{array}{ccc} F_{h}\left(F_{P_{S}},h\right) & = & h-y_{h}\\ F_{V}\left(F_{P_{T}},F_{P_{S}},F_{\theta_{T}},V\right) & = & V-y_{V}\\ F_{v_{c}}\left(F_{P_{S}},v_{c}\right) & = & v_{c}-y_{v_{c}} \end{array}$$

Let us now identify the following sets of faults:

(22) $$\begin{array}{ccc} F_{x_{1}} & = & \left\{F_{P_{T}},\;F_{P_{S}},\;F_{\theta_{T}}\right\}\\ F_{x_{2}} & = & \left\{F_{\alpha }\right\}\\ F & = & \left\{F_{x_{1}},\;F_{x_{2}}\right\} \end{array}$$

Thanks to the assumption of non-concurrent faults, it is possible to define the “active fault” as the element $F_{i}\in F$ which is non-zero and the "active fault subset" as the fault subset, among $F_{x_{1}}$ and $F_{x_{2}}$, which the active fault belongs to. Finally, let use define the *k*-th fault parameter set as:

(23) $$S_{k}:=\left\{b_{k},\eta_{k}\;:\;b_{k}\in R,\;\eta_{k}\in R\right\}$$

## 4. NLGA FDI for Singularly Perturbed Aircraft Model

This section shows novel methodological aspects arising when combining SP and NLGA for the solution of the FDI problem which can be generally stated as:

**Problem** **1.** *FDI: given the aircraft longitudinal models* (3) *and* (12) *with the fault scenario F described in* (22)*, design an FDI module with inputs $y_{1}\left(t\right)$, $y_{2}\left(t\right)$ and $u_{1},\cdots ,u_{p}$, and output the set $R=\{r_{1}^{d},\ldots ,r_{n_{R}}^{d}\}$ of $n_{R}$ binary residuals such that each fault $F_{k}$, with $k\in \left\{1,\ldots ,n_{F}\right\}$, affect a different, non-empty subset, $\Omega_{k}$, of residuals within R.*

The solution of an FDI problem can be stated in terms of a residual matrix, RM which is an $n_{R}\times n_{F}$ rectangular matrix with boolean elements where the rows list the detection residuals whereas the columns represent the fault/fault subsets. The matrix’s element RM(i,j) is equal to a logic 0 if the *i*-th detection residual is not sensitive to the *j*-th fault whereas is a boolean X∈{0,1} otherwise. The presence of the boolean *X* is motivated by the residual sensitivity to the fault with respect to the noise which will be investigated in Section 5. Finally, the subsets $\Omega_{k}$ is identified by the *k*-th column of RM.

To this end, the dynamic model of aircraft subject to faults on ADS is represented by:

(24) $$\begin{array}{c} \left[\begin{array}{c} {\dot{x}}_{1}\\ \varepsilon {\dot{x}}_{2} \end{array}\right]=\left[\begin{array}{c} g_{1,0}\left(x_{1},x_{2}\right)\\ g_{2,0}\left(x_{1},x_{2}\right) \end{array}\right]+\sum_{i=1}^{p}\left[\begin{array}{c} g_{1,i}\left(x_{1},x_{2}\right)\\ g_{2,i}\left(x_{1},x_{2}\right) \end{array}\right]u_{i} \end{array}$$

where the output is given by

(25) $$\left[\begin{array}{c} y_{1}\\ y_{2} \end{array}\right]=\left[\begin{array}{c} x_{1}\\ x_{2} \end{array}\right]+\left[\begin{array}{c} \phi_{1}\left(F_{x_{1}}\right)\\ \phi_{2}\left(F_{x_{2}}\right) \end{array}\right]$$

with

(26) $$\phi_{1}\left(F_{x_{1}}\right)=\left[\begin{array}{c} F_{h}\left(F_{P_{S}},h\right)\\ F_{V}\left(F_{P_{T}},F_{P_{S}},F_{\theta_{T}},V\right)\\ F_{v_{c}}\left(F_{P_{S}},v_{c}\right) \end{array}\right]\;\phi_{2}\left(F_{x_{2}}\right)=\left[\begin{array}{c} F_{\alpha }\\ 0 \end{array}\right]$$

Fault os output can be seen as input faults by means of the method proposed in [34] and here briefly reported: $\nu_{k}\geq 1$ equivalent and simultaneous input faults, $f_{k,i}\left(i=1,\ldots ,\nu_{k}\right)$, are introduced in place of the non concurrent output faults $F_{k}$ $\forall k\in \{1,\ldots ,n_{F}\}$. Given the *k*-th scalar output $y_{k}$ of (24), substitute $y_{k}$ to its relative state $x_{k}$ in the dynamic system (24) and identify the $\nu_{k}\geq 1$ different functions, $\phi_{k,i}\left(y_{k}\right)$ with $i\in \{1,\cdots ,\nu_{k}\}$, containing the term $y_{k}$. Define the equivalent input fault as $f_{k,i}:=\phi_{k,i}\left(x_{k}\right)$-$\phi_{k,i}\left(y_{k}\right)$ and its input vector as $l_{k,i}$. Whenever the *k*-th sensor fault occurs, i.e., $F_{k}\neq 0$, all associated input faults $f_{k,i}\left(i=1,\ldots ,\nu_{k}\right)$ will become non-zero:

(27) $$\begin{array}{c} \left[\begin{array}{c} {\dot{y}}_{1}\\ {\dot{y}}_{2} \end{array}\right]=\left[\begin{array}{c} g_{1,0}\left(y_{1},y_{2}\right)\\ g_{2,0}\left(y_{1},y_{2}\right) \end{array}\right]+\sum_{i=1}^{p}\left[\begin{array}{c} g_{1,i}\left(y_{1},y_{2}\right)\\ g_{2,i}\left(y_{1},y_{2}\right) \end{array}\right]u_{i}+\sum_{k=1}^{n_{F}}\sum_{i=1}^{\nu_{k}}\left[\begin{array}{c} l_{1_{k,i}}\left(y_{1},y_{2}\right)\\ l_{2_{k,i}}\left(y_{1},y_{2}\right) \end{array}\right]f_{k,i} \end{array}$$

where the terms $l_{1_{k,i}}$ and $l_{2_{k,i}}$ represent the input distributions of mathematical sensor faults. Let us define with the term $f_{k}$ the set of input faults associated to the sensor fault $F_{k}$:

(28) $$f_{k}:={\left\{f_{k,1},\cdots ,f_{k,\nu_{k}}\right\}}^{T}$$

The set of equivalent mathematical faults associated to F is given by the set of vectors $f_{k}$, for $k=1,\cdots ,n_{F}$, and is denoted by f:

(29) $$f:=\{f_{k},\;k=1,\cdots ,n_{F}\}$$

Finally, define with $l_{k,i}$ the mathematical fault vector field for the overall system, i.e., $l_{k,i}={\left[l_{1k,i}^{T},l_{2k,i}^{T}\right]}^{T}$ and with $l_{k}$ the vector field collecting all the input vector fields relative to the set $f_{k}$:

(30) $$l_{k}:=\left\{l_{k,1},\cdots ,l_{k,\nu_{k}}\right\}$$

Before introducing the solution to the FDI problem rewrite the system (27) as:

(31) $$\begin{array}{c} \dot{\xi }=g_{0}\left(\xi \right)+\sum_{i=1}^{p}g_{i}\left(\xi \right)u_{i}+\sum_{k=1}^{n_{F}}l_{k}\left(\xi \right)f_{k}\\ \chi =h(\xi ) \end{array}$$

where

(32) $$\begin{array}{c} \xi =\left[\begin{array}{c} y_{1}\\ y_{2} \end{array}\right],\;h\left(\xi \right)=\xi ,\;g_{0}\left(\xi \right)=\left[\begin{array}{c} g_{1,0}\left(\xi \right)\\ g_{2,0}\left(\xi \right) \end{array}\right],\;g_{i}\left(\xi \right)=\left[\begin{array}{c} g_{1,i}\left(\xi \right)\\ g_{2,i}\left(\xi \right) \end{array}\right] \end{array}$$

Im case of of fully measured state, i.e., h(ξ)=ξ, the NLGA necessary conditions for solving the FDI problem are expressed by the following algorithm [31]:

1. given a set $s\subseteq \{1,\cdots ,n_{F}\}$
2. given the fault set F, define the subset $F_{s}\subseteq F$ with $F_{s}:=\left\{F_{k},\;\;k\in s\right\}$ and the generalized disturbance $D_{s}=F\backslash F_{s}$;
3. given the equivalent fault set f associated to F, define the subset $f_{s}\subseteq f$ associated to $F_{s}$, i.e., $f_{s}:=\left\{f_{k},\;\;k\in s\right\}$, and the generalized disturbance $d_{s}=f\backslash f_{s}$ associated to $D_{s}$;
4. associate to the sets $f_{s}$ and $d_{s}$ their relative input vector fields $l_{s}$ and $p_{s}$ respectively:
  $$\begin{array}{ccc} l_{s} & :\;= & \left\{l_{k},\;\;k\in s\right\}\\ p_{s} & :\;= & \left\{l_{k},\;\;k\in \left(\left\{1,\cdots ,n_{F}\right\}\backslash s\right)\right\} \end{array}$$
5. if $l_{s}\notin {\bar{p}}_{s}$ the generalized faults set $f_{s}$ is detectable and a suitable change of coordinate can be determined.

**Property** **1.** *Given the system* (27) *with the fault scenario* (22)*, an NLGA study reveals that, defined $F_{s}=F\backslash \left\{F_{x_{2}}\right\}$ then $l_{s}={\bar{p}}_{s}$ thus implying that the necessary conditions for the solution of the FDI problem are not fulfilled for the isolation of fault affecting the air (pressure and temperature) ports and the angle of attack vane sensor.*

On the other hand, the sufficient conditions are expressed by the existence of two coordinate changes, in state and output spaces, Φ(ξ) and Ψ(χ) respectively, which consist in a surjection $\Psi_{1}$ and a function $\Phi_{1}$ such that ${\bar{p}}_{s}^{⊥}\cap span\left\{dh\right\}=span\left\{d\left(\Psi_{1}\;○\;h\right)\right\}$ and ${\bar{p}}_{s}^{⊥}=span\left\{d\Phi_{1}\right\}$, where:

(33) $$\left\{\begin{array}{ccc} \Phi (\xi ) & = & \left(\begin{array}{c} {\bar{\xi }}_{1}\\ {\bar{\xi }}_{2} \end{array}\right)=\left(\begin{array}{c} \Phi_{1}\left(\xi \right)\\ C_{2}h\left(\xi \right) \end{array}\right)\\ \Psi (\chi ) & = & \left(\begin{array}{c} {\bar{\chi }}_{1}\\ {\bar{\chi }}_{2} \end{array}\right)=\left(\begin{array}{c} \Psi_{1}\left(\chi \right)\\ C_{2}\chi \end{array}\right) \end{array}\right.$$

are (local) diffeomorphisms, whilst $C_{2}$ is a selection matrix, *i.e.* its rows are a subset of the rows of the identity matrix. If the sufficient conditions are verified then, by using the new (local) state and output coordinates $\left(\bar{\xi },\bar{\chi }\right)$, the system (31) is transformed as follows:

(34) $$\begin{array}{c} {\dot{\bar{\xi }}}_{1}={\bar{n}}_{1}\left({\bar{\xi }}_{1},{\bar{\xi }}_{2}\right)+\sum_{i=1}^{p}{\bar{g}}_{1,i}\left({\bar{\xi }}_{1},{\bar{\xi }}_{2}\right)u_{i}+{\bar{l}}_{1}\left({\bar{\xi }}_{1},{\bar{\xi }}_{2}\right)\;f_{s}\\ {\dot{\bar{\xi }}}_{2}={\bar{n}}_{2}\left({\bar{\xi }}_{1},{\bar{\xi }}_{2}\right)+\sum_{i=1}^{p}{\bar{g}}_{2,i}\left({\bar{\xi }}_{1},{\bar{\xi }}_{2}\right)u_{i}+{\bar{l}}_{2}\left({\bar{\xi }}_{1},{\bar{\xi }}_{2}\right)\;f_{s}+{\bar{p}}_{2}\left({\bar{\xi }}_{1},{\bar{\xi }}_{2}\right)\;d_{s}\\ {\bar{\chi }}_{1}={\bar{h}}_{1}\left({\bar{\xi }}_{1}\right)\\ {\bar{\chi }}_{2}={\bar{\xi }}_{2} \end{array}$$

with $l_{s_{1}}\left(\bar{\xi }\right)$ not identically zero. As described in [31], the subsystem ${\bar{\xi }}_{1}$ in Equation (34) is locally weakly observable so the detection residual associated to $f_{s}$ and thus to $F_{s}$ is given by:

(35) $$\begin{array}{c} {\dot{\hat{\bar{\xi }}}}_{1}={\bar{n}}_{1}\left({\hat{\bar{\xi }}}_{1},{\bar{\chi }}_{2}\right)+\sum_{i=1}^{p}{\bar{g}}_{1,i}\left({\hat{\bar{\xi }}}_{1},{\bar{\chi }}_{2}\right)u_{i}+Kr\\ \;\;\;r={\bar{\chi }}_{1}-h_{1}\left({\hat{\bar{\xi }}}_{1}\right) \end{array}$$

Finally, for each vectorial residual r, define a positive scalar function $r=\sqrt{r^{T}Wr}$ where W is a positive weights matrix, a positive real constant $r^{th}$ and a boolean variable $r^{d}$ defined as:

(36) $$r^{d}=\left\{\begin{array}{cc} 0\; & r\leq r^{th}\\ 1\; & r>r^{th} \end{array}\right.$$

### NLGA Combined with Singular Perturbations

This Section contains the main results of this work and shows how the FDI problem can be solved by means of the coordinated use of the SP jointly with the standard NLGA.

**Theorem** **1.** *Given the system* (24) *and for each k-th fault, there exist a subset of $S_{k}$ such that it is possible to approximate the actual dynamics of the aircraft* (24) *by its associated reduced and boundary layer models.*

**Proof.**  Let us assume that the aircraft (24) is equipped with a controller fulfilling Hypotheses 1 and 2 and define the eigenvalues of the system, affected by the *k*-th fault $F_{k}$, as $\lambda_{i}\left(\nu_{k}\right)$ with i=1,⋯,n and $\nu_{k}=\left(b_{k},\eta_{k}\right)\;\in S_{k}$. Then, as stated in [38,39] and thanks to the smoothness of the system (24) and its control system and based on their Lipschitz features, for any $\epsilon_{k}>0$ it is possible to find a sufficiently small subset $S_{k}^{⋆}\subseteq S_{k}$, possibly dependent on $\epsilon_{k}$, such that the eigenvalue of the closed loop system (24) are such that

$$\left|\right|\lambda_{i}\left({\bar{\nu }}_{k}\right)-\lambda_{i}\left(\nu_{k}\right)\left|\right|<\epsilon_{k}\;\forall \;\nu_{k}\;\in S_{k}^{⋆},\;\;i=1,\cdots ,n$$

where ${\bar{\nu }}_{k}=\left(0,1\right)$ indicates the vector of the nominal parameters (fault free condition). For each fault parameter in the set $S_{k}^{⋆}$, the model approximation is valid because the eigenvalues remain close the nominal ones so that the stability as well as the time separation properties can be kept valid.

On the base of the Theorem 1 the actual aircraft dynamics (24) is approximated by its reduced and boundary layer models:

(37) $$\begin{array}{c} \left[\begin{array}{c} {\dot{\bar{x}}}_{1}\\ x_{2}^{\prime } \end{array}\right]=\left[\begin{array}{c} g_{1,0}\left({\bar{x}}_{1},x_{2_{M}}\right)\\ g_{2,0}\left({\bar{x}}_{1},x_{2}\right) \end{array}\right]+\sum_{i=1}^{p}\left[\begin{array}{c} g_{2,i}\left({\bar{x}}_{1},x_{2_{M}}\right)\\ g_{2,i}\left({\bar{x}}_{1},x_{2}\right) \end{array}\right]u_{i}\\ \left[\begin{array}{c} y_{1}\\ y_{2} \end{array}\right]=\left[\begin{array}{c} {\bar{x}}_{1}\\ x_{2} \end{array}\right]+\left[\begin{array}{c} \phi_{1}\left(F_{x_{1}}\right)\\ \phi_{2}\left(F_{x_{2}}\right) \end{array}\right] \end{array}$$

where $x_{2_{M}}=M\left({\bar{x}}_{1},u_{1},\cdots ,u_{p}\right)$ is an isolated root of

(38) $$0=g_{2,0}\left({\bar{x}}_{1},x_{2}\right)+\sum_{i=1}^{p}g_{2,i}\left({\bar{x}}_{1},x_{2}\right)u_{i}$$

The fault mapping procedure [34] applied to (37) leads to:

(39) $$\begin{array}{c} \left[\begin{array}{c} {\dot{\bar{y}}}_{1}\\ y_{2}^{\prime } \end{array}\right]=\left[\begin{array}{c} g_{1,0}\left({\bar{y}}_{1},y_{2_{M}}\right)\\ g_{2,0}\left({\bar{y}}_{1},y_{2}\right) \end{array}\right]+\sum_{i=1}^{p}\left[\begin{array}{c} g_{2,i}\left({\bar{y}}_{1},y_{2_{M}}\right)\\ g_{2,i}\left({\bar{y}}_{1},y_{2}\right) \end{array}\right]u_{i}+\sum_{k=1}^{n_{1}}\sum_{i=1}^{\nu_{k}}\left[\begin{array}{c} l_{1_{k,i}}\left({\bar{y}}_{1},y_{2_{M}}\right)\\ l_{2_{k,i}}\left({\bar{y}}_{1},y_{2}\right) \end{array}\right]f_{k,i}+\\ +\sum_{k=n_{1}+1}^{n_{F}}\sum_{i=1}^{\nu_{k}}\left[\begin{array}{c} 0\\ l_{2_{k,i}}\left({\bar{y}}_{1},y_{2}\right) \end{array}\right]f_{k,i} \end{array}$$

where $y_{2_{M}}=M\left({\bar{y}}_{1},u_{1},\cdots ,u_{p}\right)$. The following properties have been obtained by comparing the systems (27) and (39):

1. the fault set $F_{x_{2}}$ does not affects the equations relative to ${\bar{y}}_{1}$, see Equation (39);
2. the whole fault vector F affects the equations relative to $y_{2}$ and ${\bar{y}}_{2}$, see Equations (27) and (39);
3. the vector ${\bar{y}}_{1}$ represents a constant for equations relative to ${\bar{y}}_{2}$, see Equation (39);
4. the fault set $F_{x_{2}}$ does not affects the algebraic equations relative to $x_{2_{M}}$.

These properties are exploited in the following lemmas:

**Lemma** **2.** *Given the system* (39) *with the fault scenario* (22)*, taken $F_{s}=F_{x_{1}}$ the surjection $\Psi_{1}\left(\xi \right)={\bar{y}}_{1}$ and the function $\Phi_{1}\left(\xi \right)={\bar{y}}_{1}$ are such that:*

$$span\left\{d\Psi_{1}\left(\xi \right)\right\}=span\left\{d\Phi_{1}\left(\xi \right)\right\}\subseteq {\bar{p}}_{s}^{⊥}$$

**Proof.**  Given the system (39) with the fault scenario (22), taken $F_{s}=F_{x_{1}}$ and determined $D_{s}=F_{x_{2}}$, the $d_{s}$ is defined as $d_{s}:\;=\left\{f_{k},\;\;k\in \left\{n_{1}+1,\cdots ,n_{F}\right\}\right\}$ with its associated input vector field defined $\forall \;i\in \{1,\cdots ,\nu_{k}\}$ and $\forall \;k\in \{n_{1}+1,\cdots ,n_{F}\}$ as:

$$p_{s}:\;=\left\{\left[\begin{array}{c} 0\\ l_{2_{k,i}}\left({\bar{y}}_{1},y_{2}\right) \end{array}\right]\right\}$$

In conclusion, the involutive closure of $p_{s}$ is such that $span\left\{d{\bar{y}}_{1}\right\}\subseteq {\bar{p}}_{s}^{⊥}$ because, thanks to the Singular Perturbation approximation, the first $n_{1}$ components of each vector field in $p_{s}$ are null. ☐

**Lemma** **3.** *Given the system* (39) *with the fault scenario* (22)*, given F and $F_{x_{1}}$ as in Property 1 and defined the input vector fields relative to their generalized disturbance, p and $p_{x_{1}}$ respectively, then ${\bar{p}}^{⊥}⊃{\bar{p}}_{x_{1}}^{⊥}$.*

**Proof.**  The combined used of Lemma 2 and SP shows that the projection of ${\bar{p}}^{⊥}$ on ${\bar{y}}_{1}$ has components that the projection of ${\bar{p}}_{x_{1}}^{⊥}$ has not. As consequence, a coordinate change can be found to design a residuals that is independent from $F_{\alpha }$. ☐

**Lemma** **4.** *Given the system* (27) *and its singularly perturbed approximation* (39)*, then the active fault subset is isolable.*

**Proof.**  Two different residuals are designed on the base of Property 1 and Lemma 2, $r_{1}^{d}$ and $r_{2}^{d}$. The first one, $r_{1}^{d}$, is originated by $\Phi_{1}\left(\xi \right)=\Psi_{1}\left(\xi \right)=y_{1}$ and the second one, $r_{2}^{d}$, exploits the singular perturbed coordinate change $\Phi_{1}\left(\xi \right)=\Psi_{1}\left(\xi \right)={\bar{y}}_{1}$. The residual $r_{1}^{d}$ and $r_{2}^{d}$ are organized in a residual matrix, depicted in Table 1, where the second row results to be fundamental to isolate the active fault subset. ☐

**Remark** **2.** A study of Table 1 reveals that the elimination of the residual $r_{2}^{d}$, introduced thanks to the adoption of the SP approximation, make the the isolation the faults in $F_{x_{1}}$ and the fault on the vane sensor, $F_{\alpha }$, structurally impossible. Furthermore, can be demonstrated that Lemma 4 is valid also when considering the whole aircraft dynamics (comprehensive of lateral and directional motions) also in presence of faults affecting the side-slip angle vane sensor (considered as a further element of the fault set $F_{x_{2}}$).

## 5. Simulation Results

**Figure 4:**
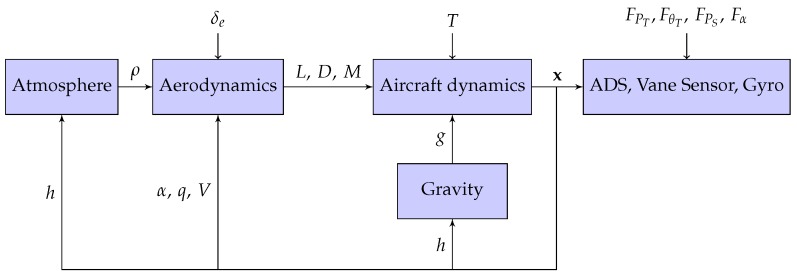
Simulator structure.

The results presented in this paper have been obtained in simulation. The simulation benchmark is represented by a general aviation aircraft for which technical reports are available in literature. In particular NASA Technical Notes [40,41,42] describe the aerodynamics of the aircraft and the propeller. Figure 4 graphically depicts the blocks diagram details composing the simulator:

- Aircraft Dynamics: The dynamics of the aircraft, seen as a rigid body with six degree of freedom, is altered by torques and forces inducing accelerations which, integrated two times, generate speeds and positions. Euler angles describes the attitude of the aircraft;
- Aerodynamics: The NASA reference [40] graphically reports the aerodynamic coefficients of lift *L*, drag *D* and pitch momentum *M* which are functions of the angle of attack α and the thrust coefficient $T/(q_{d}S)$. The simulator implements these coefficients by means of look-up tables;

During the simulations the updating of both the air density, ρ, and the gravity acceleration modulus, *g*, are performed by implementing the reference [37,43] respectively.

The simulation of the sensor suite follows what stated in [44,45]:

- the pitch rate is provided by means of one gyroscope of an IMU (Inertial Measurement Unit). The measurement errors are comprehensive of non unitary scale factor, alignment error (random), g-sensitivity, additive white noise and gyro drift;
- Air Data System (ADS):
  - The true air-speed is affected by calibration error of the differential pressure sensor plus additive white noise;
  - The altitude measurement is corrupted by calibration error of the static pressure port plus additive white noise;
  - The attack angle vane sensor is influenced by calibration errors plus additive white noise.

A detailed description of the measurements used by the considered system can be found in [14].

### 5.1. Sensitivity Analysis

Uncertainties in model parameters influence the NLGA results in terms of decoupling from the disturbances $d_{s}$, which is not more perfect, and reduce the sensitivity of ${\bar{\xi }}_{1}$ with respect to $f_{s}$ thus making the fault detection and isolation harder. It is rather intuitive that, due to the residual sensitivities and model uncertainties, for each fault $F_{k}$, different fault severities could identify actual residual subsets $\Omega_{k}$ that are contained in those listed in Table 1.

In particular, for each $k=1,\cdots ,n_{F}$, there exist:

- an *undetectable fault parameter set* defined as $S_{k_{\mathrm{und}}}\subseteq S_{k}^{⋆}$ such that $\forall \;\nu_{k}\in S_{k_{\mathrm{und}}}$ each binary residual $r_{i}^{d}=0$, with $i=1,\cdots ,n_{R}$, so leading to $\Omega_{k}=\left\{\emptyset \right\}$ for each $k=1,\cdots ,n_{F}$;
- a *detectable fault parameter set*, $S_{k_{\det}}\subseteq S_{k}^{⋆}$, defined as the fault set for which the detection is guaranteed (but not the isolability). In particular, there exist a couple of fault parameters $\nu_{i}$ and $\nu_{j}$ with $i,\;j\in \{1,\cdots ,n_{F}\}$ with i≠j, respectively belonging to $S_{i_{\det}}$ and $S_{j_{\det}}$ for which the residuals configurations $\Omega_{i}$ and $\Omega_{j}$ are non-empty but equal thus not providing the isolability;
- an *isolable fault parameter set*, $S_{k_{\mathrm{isol}}}\subseteq S_{k}^{⋆}$, identified as the fault parameter set such that $\forall \;\nu_{k}\in S_{k_{\mathrm{isol}}}$ the residuals configuration $\Omega_{k}$ is unique. In particular, for each couple of fault parameters $\nu_{i}$ and $\nu_{j}$ with $i,\;j\in \{1,\cdots ,n_{F}\}$ with i≠j, respectively belonging to $S_{i_{\mathrm{isol}}}$ and $S_{j_{\mathrm{isol}}}$ the residuals configurations $\Omega_{i}$ and $\Omega_{j}$ are non-empty and different.

**Figure 5:**
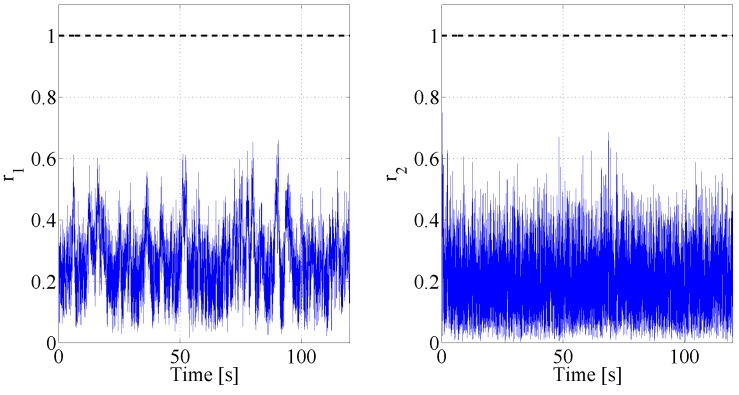
Residuals and thresholds: behaviour in absence of faults.

Given the analogical vector residual $r_{i}$ and an associated comparison threshold $r_{i}^{th}$, the binary residual $r_{i}^{d}>0$ is obtained by means of the following law

(40) $$r_{i}^{d}:=\left\{\begin{array}{cc} 1 & \sqrt{\left|\right|r_{i}^{T}W_{i}r_{i}\left|\right|}>r_{i}^{th}\\ 0 & \sqrt{\left|\right|r_{i}^{T}W_{i}r_{i}\left|\right|}\leq r_{i}^{th} \end{array}\right.$$

where the elements of the matrices $W_{i}=\frac{1}{\sqrt{\dim(r_{i})}}\mathrm{diag}\left(\left[w_{i,1},\cdots ,w_{i,\dim(r_{i})}\right]\right)$ have been defined in the following way:

(41) $$\underset{F_{s}=\left\{\emptyset \right\}}{\sup}w_{i,j}^{2}r_{i,j}^{2}={\left(r^{th}\right)}^{2}$$

for each $j\in \{1,\cdots ,\dim\left(r_{i}\right)\}$ where $\dim(r_{i})$ indicates the dimension of the vector $r_{i}$. Table 2 reports the values given to the weight matrices. As result, the two residuals live under the threshold $r^{th}$ (set equal to 1 in this paper) when in absence of faults, see Figure 5.

**Figure 6:**
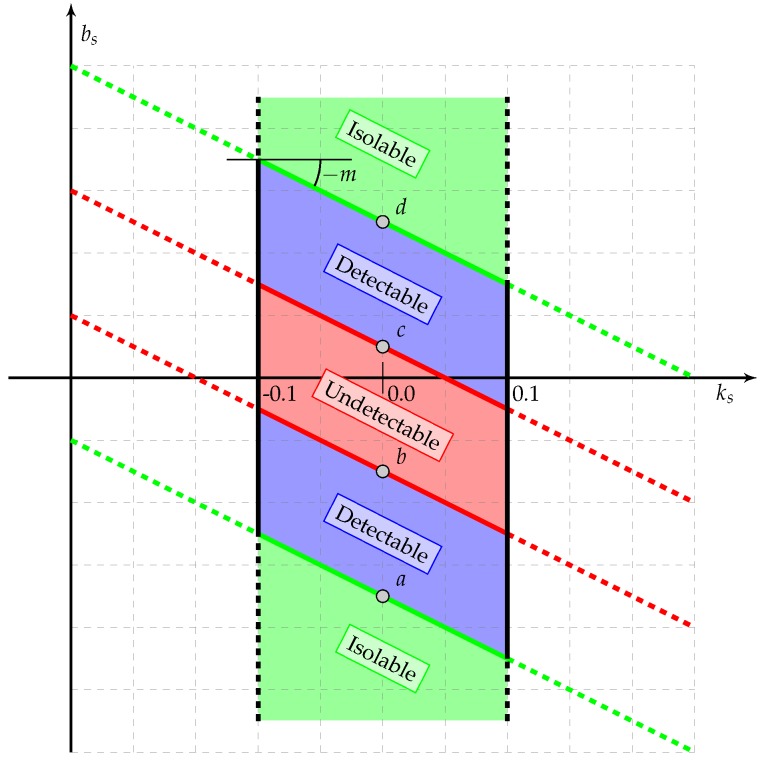
Representation of the fault parameter sets.

For each $k\;\in \;\{1,\cdots ,n_{F}\}$ the sets $S_{k_{\mathrm{und}}}$, $S_{k_{\det}}$ and $S_{k_{\mathrm{isol}}}$ have been numerically determined in presence of the most common uncertain quantity in standard general aviation aircraft (mass, inertia and drag coefficients) and simulated as a random variable with uniform distribution in the set of ±5% of the corresponding nominal parameter. Table 3 and Table 4 and respectively list the nominal parameters, exploited by the FDI module, and their minimum and maximum values assumed into the plant. Table 5 lists the resulting fault sets whose bounds, shown in Figure 6, are obtained by assuming a slope modification $k_{s}\in \left[-0.1,\;\;0.1\right]$. All the results presented in this work have been obtained by means of an extended Monte-Carlo campaign covering all the cruise flight envelope (unitary positive load factor and air-speed up to the manoeuvre speed).

**Remark** **3.** The faults considered in this paper are corrupting the sensors and do not directly affect the plant, i.e., they are not system faults which modify the plant structure or the plant parameters. Anyway the fault have a secondary effect on the robustness of the proposed method because the aerodynamic coefficients, exploited by the FDI module, are obtained from look-up tables which entries contain also the angle of attack. This implies that erroneous values of α lead to erroneous values of the lumped parameters exploited to isolate the faults. All the simulation results are comprehensive of this phenomenon.

**Remark** **4.** *The concept of the fault sets have been introduced to describe the behaviour of the detection and isolation logic in presence of faults modelled as sum of biases and slope modifications, i.e., $F_{s}=b_{s}+\left(k_{s}-1\right)s$. The sets are given to show how the combination of $b_{s}$ and $k_{s}$ influences the detection and isolation results: given $k_{s}$ the faults can be undetectable, detectable or isolable are functions of the value of $b_{s}$. The identified fault sets are realistic due to the high fidelity of the simulator. As example the maximum tolerable fault on the static port is fixed by the vertical separation of the air traffic. In particular, the vertical separation is 75 m and a fault of ±690 Pa would lead to an error of about ±60 m [35]. The minimum isolable fault size identified in this paper is about ±155 Pa which is in line with the mentioned limits. Moreover, the aeronautical standard allows for a maximum tolerable error of ±4 knots on the airspeed. The fault size of ±55 Pa on the total air pressure port leads to an airspeed variation of less than ±2 knots while the fault on the static pressure port of ±155 Pa leads to an error of about ±4.5 knots. Finally a fault of about ±7 deg on the total air temperature port leads to an erroneous airspeed of about ±1.5 knots.*

### 5.2. Fault Isolation Performance

**Figure 7:**
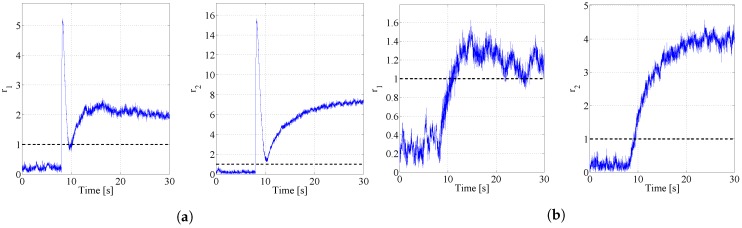
Residuals in case of faults on the static and total air pressure ports: (**a**) Fault on $P_{S}=-150.0$ Pa; (**b**) Fault on $P_{T}=-87.9$ Pa.

To complete the dissertation about the fault detection and isolation, this work ends with the presentation of some single simulation runs in which the aircraft has been set to a cruise at speed 116 knots and altitude of 3280 fts, for a nominal static air pressure and temperature of 89,875 Pa and 8 °C respectively. The trim conditions are

$$\begin{array}{c} \alpha =2.1\;\left[\deg\right];\;q=0\;\left[\deg/s\right];\;T=1469.6\;\left[N\right];\;\delta_{e}=0.22\;\left[\deg\right]. \end{array}$$

whereas the faults dynamics has been characterized by a time constant $\Delta_{t}$ of 1 s. Figure 7 report the behaviours of the two detection residuals in presence of faults on the static air pressure port (Figure 7a) and on the total air pressure port (Figure 7b). Both the residuals, in accordance with their design criterion, overcome their threshold thus identifying the configuration of the residual matrix. The graphs in Figure 7a clearly show a quasi impulsive behaviour due to the variation of the rate of climb induced by a fault on the static pressure port: the rate of climb is a low pass filtered version of the static pressure first time derivative indeed. Once this effect disappears the asymptotic value of the residuals is due to the erroneous values of the airspeed. This impulsive behaviour is not present in Figure 7b where the aircraft is affected by faults on the total air pressure port because this measurement does not influence the rate of climb.

**Figure 8:**
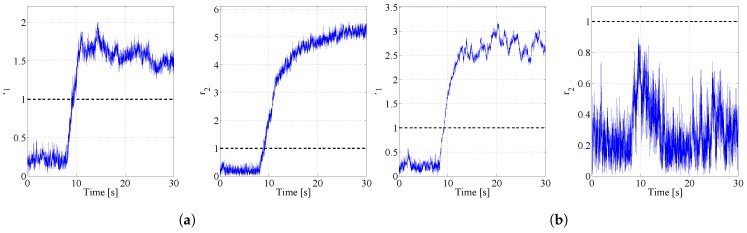
Residuals in case of faults on the total air temperature ports and the angle of attack vane sensor: (**a**) Fault on $\theta_{T}=-15.5$ °C; (**b**) Fault on α=0.34 deg.

The composition of the residual matrix is replicated in the behaviour of the analogical residuals depicted in Figure 7a and Figure 8b.

In conclusion, Table 6 reports the result of a 10,000-runs Monte-Carlo simulation in which, with respect to the sets described in Table 6, the faults have been randomly generated with a uniform distribution. The following dimensionless indexes constitute the meter for evaluating the performance of the FDI module:

- Missed Fault Rate (MFR): division of the number of not detected faults over the total number of simulated faults;
- False Alarm Rate (FAR): ratio of the number of faults which have been detected over the number of simulations performed in absence of faults;
- Detection Rate (DR): number of faults that have been detected over the total number of simulations in presence of faults;
- Isolation Rate (IR): division of the number of faults which have been correctly isolated over the total number of simulations in presence of faults;
- Wrong Isolation Rate (WIR): ratio of the number of faults which have been wrongly isolated over total number of simulations in presence of faults.

The values reported in Table 5 indicate a promising effectiveness of the proposed solution. With respect to the isolability of the faults affecting the total air pressure port, there is an inherent difficulty in the isolation of the faults affecting the static pressure port. In particular, a fault increasing $P_{S}$ produces two counteracting effects: on one hand it increases the estimated airspeed $\hat{V}$ but on the other it reduces the estimated air density $\hat{\rho }$ such that the final variation of the pressure dynamic $q_{d}=\frac{1}{2}\hat{\rho }{\hat{V}}^{2}$ is, in modulus, smaller than the variation due to a fault, with the same magnitude, but affecting the total air pressure port. Since the pressure port faults reveal themselves by means of the dynamic pressure, this phenomenon makes easier the isolation of the faults on the total air pressure. The same conclusions can be obtained about the isolability of the faults affecting the total air temperature port. Furthermore, thanks to the structure of the detection residuals the isolation of faults affecting the vane sensor α is almost perfect (WIR 2%): the second detection residual, $r_{2}$, is theoretically independent from fault on α thus reducing the possibility of confusing the residual configuration. Values of WIR different from zero, for $F_{\alpha }$, are due to the presence of model uncertainties which makes the decoupling not perfect. Finally the WIR values relative to fault in $F_{x_{1}}$ are also due to the residual structure: for some flight conditions, these faults may force only the first residual $r_{1}$ out of its threshold thus inducing a wrong isolation.

## 6. Conclusions

Given a general aviation aircraft affected by faults on the air data system, this paper proposed a novel approach for solving the Fault Detection and Isolation problem. The cornerstone of the proposed approach stays in the coordinated use of the NonLinear Geometric Approach and the Singular Perturbation theory and represents a suitable solution for designing Fault Detection and Isolation schemes for all that plants whose dynamics can be described by two-time scales. This work demonstrated that, by means of the proposed approach, fault affecting the angle of attack and those affecting the air (both pressure and temperature) probes can be correctly detected and isolated. An aircraft flight simulator, based on high fidelity experimental data of one real general aviation aircraft, has been used as benchmark for assessing the results showed in this paper. Future works will focus on the improvement of the isolation performance by means of the introduction of further sensors.

## Figures and Tables

**Table 1:** Residual matrix for the isolation of the active fault subset.

Residual	$F_{x_{1}}$	$F_{x_{2}}$
$r_{1}^{d}$	X	X
$r_{2}^{d}$	X	0

**Table 2:** Weight matrices.

Residual	Weights Matrix
$r_{1}$	$W_{1}=\mathrm{diag}\left(\left[0,\;\;1.5625,\;\;25,\;\;1600,\;\;156.25\right]\right)$
$r_{2}$	$W_{2}=\mathrm{diag}\left(\left[1.5625,\;\;100\right]\right)$

**Table 3:** Uncertain Parameters: mass and inertia.

Par.	Units	Minimum Value	Nominal Value	Maximum Value
m	kg	1548.5	1630	1711.5
$I_{y}$	kg·m^2^	2446.1	2574.8	2703.5

**Table 4:** Uncertain Parameters: drag coefficient, $C_{D}\left(\alpha \right)$.

α [deg]	−3.5	−1.5	0.5	2.5	4.5	6.5	8.5	10.5	12.5	14.5	16.5	18.5
**min.** $C_{D}$	0.03325	0.0285	0.0285	0.03325	0.04275	0.057	0.08075	0.114	0.152	0.209	0.2755	0.361
**nom.** $C_{D}$	0.035	0.03	0.03	0.035	0.045	0.06	0.085	0.12	0.16	0.22	0.29	0.38
**max.** $C_{D}$	0.03675	0.0315	0.0315	0.03675	0.04725	0.063	0.08925	0.126	0.168	0.231	0.3045	0.399

**Table 5:** Sensor Fault Sets.

Variable	Units	m	*a*	*b*	*c*	*d*
$P_{S}$	Pa	89,875	−150.0	−20.0	25.0	155.0
$P_{T}$	Pa	91,876	−51.5	−13.0	14.5	55.5
$\theta_{T}$	°C	10	−7.3	−2.0	2.0	7.2
α	deg	2.1	−0.22	−0.05	0.07	0.17

**Table 6:** Monte-Carlo Simulation: sensor faults.

Var.	$b_{s_{\min}}$	$b_{s_{\max}}$	MFR	FAR	DR	IR	WIR
$P_{S}$	−1921	707	1.71	2.98	98.29	88.58 ^1^	9.70 ^1^
$P_{T}$	−284	1667	1.41	3.01	98.59	95.85 ^1^	2.74 ^1^
$\theta_{T}$	−71	72.5	2.79	3.12	97.21	89.90 ^1^	7.32 ^1^
α	−4.8	8	0.94	2.75	99.06	96.95	2.11

^1^ Despite the NLGA analysis clearly states that it is impossible to isolate faults belonging to $F_{x_{1}}$, the occurrence of a fault in the group $\left\{F_{P_{S}},\;F_{P_{T}},\;F_{\theta_{T}}\right\}$ can be isolated from $F_{\alpha }$ with the percentage indicated by the IR. Accordingly, the WIR indicates the percentage of test which resulted in the isolation of a fault out of the set $F_{x_{1}}$
